# Characterization and Performance Evaluation of Metakaolin-Based Geopolymer Foams Obtained by Adding Palm Olein as the Foam Stabilizer

**DOI:** 10.3390/ma15103570

**Published:** 2022-05-17

**Authors:** Qinglin Yu, Xueying Li, Zheng Wang, Jing Xue

**Affiliations:** 1 School of Civil Engineering, Harbin Institute of Technology, Harbin 150090, China; zhengwang5808@163.com; 2 Key Lab of Structures Dynamic Behavior and Control of the Ministry of Education, Harbin Institute of Technology, Harbin 150090, China; 3 Key Lab of Smart Prevention and Mitigation of Civil Engineering Disasters of the Ministry of Industry and Information Technology, Harbin Institute of Technology, Harbin 150090, China; 4 School of Architecture, Harbin Institute of Technology, Shenzhen 518055, China; jingxhitsz@163.com

**Keywords:** geopolymer foams, foam stabilizer, pore structure, water absorption, thermal conductivity

## Abstract

Geopolymer foams with different pore structures can be used in construction, water treatment, and heavy metal adsorption. The preparation of high porosity geopolymer foams using vegetable oil as a foam stabilizer is a feasible and cost-effective route. In this study, metakaolin-based geopolymer foams with hierarchical pore structures were fabricated by adding H_2_O_2_ as the foaming agent with palm olein as the foam stabilizer. The effects of H_2_O_2_ and palm olein content on the chemical features and pore structure of geopolymer foams were evaluated. Water absorption, thermal conductivity, and mechanical behaviors of geopolymer foams were also investigated. The results indicate that fatty acid salt surfactants were generated in situ in the geopolymer matrix due to the addition of palm olein. Geopolymer foams with H_2_O_2_ and palm olein addition possess a homogeneously concentrated macropore distribution. Palm olein exhibits a refining effect on intrinsic pores formed by geopolymerization. In addition, using appropriate amounts of palm olein and H_2_O_2_, geopolymer foams can achieve higher open porosity and better pore connectivity, resulting in the improvement of water absorption and thermal insulation capacity.

## 1. Introduction

Geopolymers are inorganic polymers with zeolite-like structures synthesized by the alkali activation of alumina silicate precursors, such as metakaolin (MK), fly ash, red mud, and several industrial byproducts. It is a material that possesses excellent mechanical properties [1], chemical and high-temperature resistance [2] and is available for various fields. The low carbon emissions in raw material production and preparation process means geopolymers have the potential to replace traditional high-energy-consuming materials. With a proper structural design, such as lightweighting, geopolymers can become versatile, eco-friendly materials with a low carbon footprint throughout their life cycle.

Geopolymer foams, also called lightweight or porous geopolymers, are new porous materials fabricated by introducing pores into the geopolymer matrix. Due to their characteristics as both geopolymer and porous materials, geopolymer foams have been widely investigated by researchers recently. The properties of geopolymer foams are significantly influenced by their pore characteristics, such as porosity, pore types (closed or open), connectivity, and pore size distribution [3,4,5,6]. Generally, geopolymer foams with higher porosity possess lower compressive strength, thermal conductivity, and bulk density [5]. However, the effect of different pore types on the performance of geopolymer foams is inconsistent. Compared with open pores, fine and uniform closed pores are more beneficial for improving mechanical and thermal insulation properties [3]. With elevated open porosity, the connectivity of isolated pores in the structure increases, forming irregular large open and connected pores [4]. Geopolymer foams having connected pore networks own high specific surface area and volume capacity, which make them hold significant advantages in terms of water and sound absorption, although the mechanical properties are slightly inferior [5]. In addition to the above factors, the pore size distribution significantly affects the performance of geopolymer foams [6]. Samples with wider average pore size show poorer compressive strength but may exhibit superior thermal insulation capacity if they have higher porosity. As previously described, geopolymer foams with lower pore connectivity generally have superior strength and low thermal conductivity, making them suitable for construction [7,8], thermal barrier [9,10,11], and sound insulation [12,13]. With improved pore connectivity, the porosity and specific surface area of geopolymer foams increase accordingly. It means that geopolymer foams can be more widely used in wastewater treatment (adsorption of heavy metals [14,15,16] and organic pollutants dissolved in water [17]) and the preparation of functionalized composite materials [18,19]. Therefore, the regulation and optimization of the pore structure in geopolymer foams have become one of the top research hotspots in this field.

Geopolymer foams with various pore structures can be prepared by changing the preparation method and adjusting raw materials and additives [20,21]. The main fabrication methods of geopolymer foams can be divided into four categories: (i) Direct foaming, (ii) Sacrificial templates, (iii) Additive manufacturing, and (iv) Other methods. The direct foaming method is one of the most common methods used in the preparation of geopolymer foams. Several foaming agents, Al powder [22,23,24], Si powder [25], and H_2_O_2_ [26,27] are most commonly selected to introduce gases to the geopolymer matrix. Among them, H_2_O_2_ is the most desirable for its economical, efficient, and impurity-free nature. It decomposes and releases oxygen in the matrix to develop pore structures. In order to prevent the escape of gases from the matrix and excessive merging of air bubbles, foaming agents are usually used in conjunction with foam stabilizers.

Foam stabilizers can elevate the stability of foams by strengthening the molecular structure of the pore walls and reducing the surface tension of the mixture [3]. Some surfactants or stabilizing agents, such as sodium dodecyl sulfate (SDS) [28,29], sodium lauryl ether sulfate (SLES) [5], tween80 [30], and vegetable oils [17,27,31], are often used as foaming stabilizers to prepare geopolymer foams in combination with foaming agents [5,17,27,28,29,30,31]. Compared to those prepared with other foam stabilizers, geopolymer foams that are fabricated using vegetable oils have higher porosity [32,33]. With a low cost and pollution-free production process, vegetable oil is a desirable option for foam stabilizers to achieve eco-friendly geopolymer foams. However, the types and proportions of fatty acids in vegetable oils significantly affect the pore structure of geopolymer foams. Vegetable oils high in palmitic and oleic acids are preferable for preparing geopolymer foams with high porosity and concentrated pore size distribution, such as palm olein and olive oil [27]. Palm olein is the liquid fraction of palm oil after fractionation and crystals removal [34]. It mainly contains palmitic acid (38–43%), oleic (39–45%), and linoleic acids (10–13%). The composition of palm olein makes it a more suitable foam stabilizer for preparing geopolymer foams with high open porosity than conventional vegetable oils [27].

The pore characteristics of geopolymer foams, such as pore size distribution, porosity, and connectivity, are obviously influenced by the amount of foaming agent and stabilizer used. It also further affects the macroscopic properties of geopolymer foams. The image analysis method has been employed to characterize the pore structure of geopolymer foams in published studies. However, limited by the capacity of this technique, it is difficult to obtain information about small pores, such as those smaller than 1 μm. Meanwhile, quantitative characterization of the connectivity of macropores is vital for establishing the relationship between microstructure and macroscopic properties, which are rarely reported in the existing literature. In this study, palm olein was introduced as a new foam stabilizer for geopolymer foams preparation coupled with H_2_O_2_ as the foaming agent. The chemical features of geopolymer foams with different H_2_O_2_ and palm olein content were investigated. In order to gain a comprehensive understanding of the effects of H_2_O_2_ and palm olein on different types of pores in geopolymer foams, an integrated characterization was performed by combining mercury intrusion porosimetry (MIP), image analysis, and geometric calculations. In parallel, we will attempt a quantitative description of the pore connectivity of geopolymer foams using a simplified geometric model, which was first proposed. Furthermore, the influences of H_2_O_2_ and palm olein on the water absorption, thermal conductivity, and mechanical behaviors of geopolymer foams were also examined. We hope that the work performed in the paper will provide a feasible way for the preparation and application of low carbon footprint geopolymer foams.

## 2. Materials and Methods

### 2.1. Materials

#### 2.1.1. Precursor

**Figure 1:**
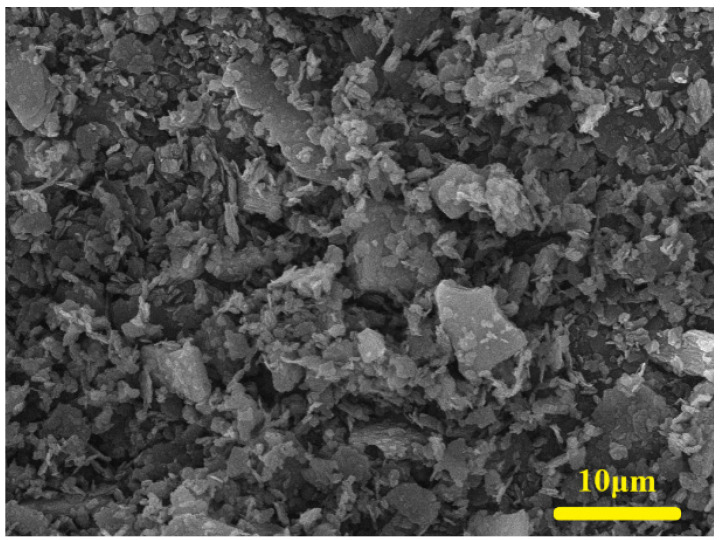
Microscopic morphology of metakaolin.

**Figure 2:**
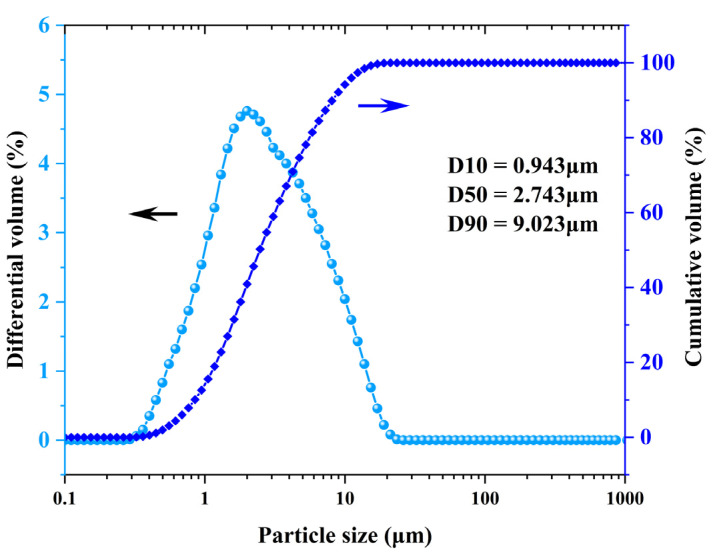
Particle size distribution of metakaolin.

Metakaolin (Calcined kaolin, SP33, BASF Corporation, Florham Park, NJ, USA), with a bulk density of 0.445 g/cm^3^, was selected as the precursor material to prepare geopolymer foams. The chemical composition was analyzed by an X-ray fluorescence spectrometer (XRF, Panalytical Axios, Almelo, The Netherlands), as listed in Table 1. Figure 1 shows the morphology of metakaolin under SEM. As can be seen from the image, most of the particles of metakaolin are smaller than 10 μm. The particle size distribution of metakaolin was analyzed by a laser particle size analyzer (BT-2001, BETTER, Dandong, China), as shown in Figure 2.

#### 2.1.2. Alkali Activator

The alkali activator was prepared by mixing aqueous sodium hydroxide with liquid sodium silicate. Sodium hydroxide (Analytical reagent, NaOH ≥ 96.0%, Dalu, Tianjin, China) is mainly used to reduce the modulus of the activator and make its viscosity suitable for mixing. The aqueous sodium silicate (molar ratio of SiO_2_/Na_2_O = 2.2, H_2_O content = 55.61%) used in this experiment was purchased from Hebei Julide Co., Ltd. (Langfang, China).

#### 2.1.3. Foaming Agent

H_2_O_2_ solution (Analytical reagent, purity 30 wt.%, Keshi, China) was used as the foaming agent. H_2_O_2_ will slowly and spontaneously decompose into water and oxygen at room temperature, according to Equation (1). The decomposition process will be more violent at elevated temperatures and in alkaline solutions.

(1) $$H_{2}O_{2\;\left(\mathrm{liquid}\right)}\to H_{2}O_{\;\left(\mathrm{liquid}\right)}+O_{2\;\left(\mathrm{gas}\right)}$$

#### 2.1.4. Foam Stabilizer

Palm olein (Food grade, Julong, Tianjin, China) was chosen as the foam stabilizer to prepare geopolymer foams with H_2_O_2_. Distinct from palm stearin, which has a high melting point [27], the melting point of palm olein is below 20 °C [34], which makes it feasible to prepare geopolymer foam with palm olein by a one-step process at room temperature without additional heat treatment. The fatty acids in palm olein are saponified with an alkali solution in the matrix. Fatty acid salts, as the surfactants, are generated in situ by the saponification.

### 2.2. Fabrication of Geopolymer Foams

Prior to the preparation of geopolymer slurry, the alkali activator, with molar ratio SiO_2_/Na_2_O/H_2_O = 1.2/1.0/15.0, was obtained by thoroughly mixing liquid sodium silicate and a 6.8 M NaOH solution (prepared by dissolving NaOH particles in tap water). The alkali activator should be sealed and placed at room temperature for more than 24 h before use.

**Figure 3:**
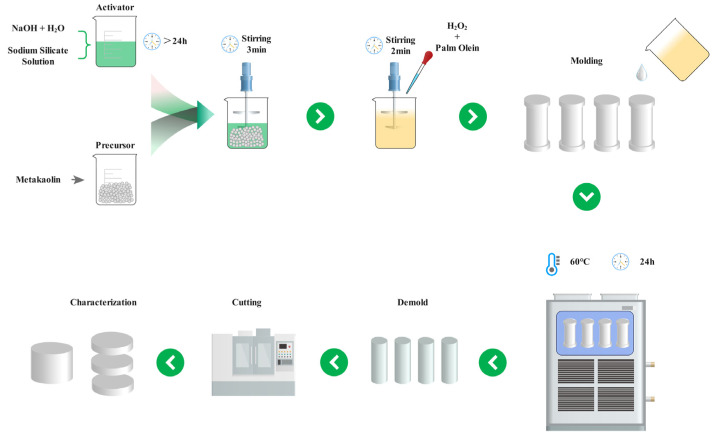
Fabrication process of geopolymer foams.

Figure 3 presents a schematic diagram of the preparation process of geopolymer foams. The geopolymer slurry was prepared by mechanical mixing of metakaolin and the alkali activator at 1000 rpm for 3 min. H_2_O_2_ and palm olein were then added to the slurry and stirred for 2 min at 1000 rpm according to the mix proportion listed in Table 2 Adding the slurry to one-third of the cylindrical plastic mold (ϕ35.0 × 85.0 mm) leaves enough space for volume expansion.

After filling the slurry, the molds were sealed with special caps and transferred to a 60 °C oven for 24 h. The samples were taken out from the dry oven and demolded after the temperature of the samples equilibrated to room temperature one day later. Cylindrical samples were cut into discs suitable for characterization using a precision cutter (SYJ-200, KeJing, Shenyang, China) with 10 μm precision. The prepared specimens were dried to constant weight in a blast drying oven at 60 °C before testing.

The synthesized geopolymer foams samples were named according to H_2_O_2_ and palm olein amounts. The letters H and P represent H_2_O_2_ and palm olein, respectively. The number following each letter represents the percentage by mass of that additive to the metakaolin. Matrix samples (H0P0) without blowing agent and foam stabilizer were also prepared in the same proportions as a reference.

### 2.3. Characterization

#### 2.3.1. Chemical Features

Fourier-transform infrared (FTIR) (Nicolet iS10, Thermo Fisher, Waltham, MA, USA) was employed to determine the functional groups and chemical bonds in geopolymer foams. The wavenumber range was 4000–400 cm^−1^ with a resolution of 2 cm^−1^. The phases compositions of geopolymer foams were evaluated using X-ray diffraction (XRD) (PANalytical Xpert3, MalvernPanalytical, Malvern, UK) with CuKα X-ray (wavelength equal to 1.54 angstrom) generated at 40 kV and 40 mA in the 2 theta range from 10 to 80. Step size and scan step time were set to 0.013° and 30 s, respectively.

#### 2.3.2. Morphology

Three imaging methods were used to characterize the morphologies of geopolymer foams. A high-resolution camera (IMX498, Sony, Tokyo, Japan) was employed to capture the full-size images of geopolymer foams. The millimeter-scale and micron-scale topography were characterized by Optical-digital microscope (DSX500, Olympus, Tokyo, Japan) and SEM (VEGA3 XMU, TESCAN, Brno, Czech Republic). The samples were fixed on a sample stage and sputtered with a 10 nm gold coating to improve conductivity prior to SEM observation. Secondary electron images of geopolymer foams were obtained by magnifying 500 times with 5 kV accelerating voltage.

#### 2.3.3. Pore Size Distribution

A MIP apparatus (AutoPore IV 9500, Micromeritics, Norcross, GA, USA) was adopted to evaluate the pore structure of geopolymer foams and the matrix sample. The pressure analysis was performed for pressure from 0.52 to 33,000 Psia, which means the measurable pore size distribution ranges from 0.005 to 350 μm. The mercury parameters used in this experiment, including contact angle and surface tension, were 130° and 485 dynes/cm, respectively. The samples were cut into approximately 4 mm × 4 mm × 4 mm pieces and vacuum dried for 4 h before testing. In this study, a powder penetrometer with a stem volume of 1.31 mL is preferred for geopolymer foams characterization.

#### 2.3.4. Porosity

Geopolymer foams contain pores ranging from nanometers to millimeters [35,36]. The total open porosity (${Ø}_{total}$) of each sample was calculated by the following Equation (2):

(2) $${Ø}_{total}=\left(1-\frac{\rho_{bulk}}{\rho_{skeletal}}\right)\times 100\%$$

where $\rho_{bulk}$ is the bulk density calculated by the geometric method (ratio of weight to volume). A caliper with an accuracy of ±0.02 mm was used to measure the dimensional data of samples. $\rho_{skeletal}$ is the skeletal density deduced from data collected by MIP and directly reported in the result files.

#### 2.3.5. Pore Shape

*Roundness* is used to evaluate the irregularity of pores [4,37]. It can be calculated by Equation (3):

(3) $$Roundness=\frac{P^{2}}{4\pi A}$$

where *A* and *P* are the area and diameter of the pore, respectively. These two parameters can be measured by ImagePro Plus software (v6.0, Media Cybernetics, Maryland, USA) from the binarized cross-sectional image of each sample. Equation (3) is the equation adopted by the ImagePro Plus software to calculate the *roundness*. An alternative method of calculating *roundness* used by some researchers [38] is given in Equation (4), whose values are reciprocal to that of Equation (3).


(4) $$Roundness^{1}=\frac{4\pi A}{P^{2}}$$

In this study, the two-dimensional pore data were acquired by ImagePro Plus software. For the convenience of data processing, Equation (3) was chosen for *roundness* calculation.

#### 2.3.6. Water absorption and thermal conductivity

The water absorption of geopolymer foams was conducted according to ASTM C642. Three specimens with a diameter of 35 mm and a thickness of 10 mm were tested for each sample. An average value of these test results was taken. The water absorption can be calculated by Equation (5).

(5) $$Water\;absprption\;\left(\%\right)=\frac{m_{saturated}-m_{dry}}{m_{dry}}\times 100\%$$

where $m_{saturated}$ is the mass of water-saturated specimen (g), $m_{dry}$ is the mass of dry specimen (g).

The thermal conductivity of geopolymer foams at room temperature was determined using a hot wire thermal conductivity analyzer (TC3000E, XIATECH, Xi′an, China). Six specimens of the same size as the water absorption test were used to measure the thermal conductivity of geopolymer foams. The hot wire probe should be in good contact with the samples during the measurement. Thermal conductivity testing three times for each set of specimens to ensure reproducibility and accuracy.

#### 2.3.7. Compressive Strength

The compressive strength of matrix and geopolymer foams was determined three days after sample fabrication using a universal mechanical testing machine (YAW-300D, HRJ, Jinan, China) with a loading speed of 2400 N/s. The final value of compressive strength is the average result of 3 specimens with dimensions of ϕ35 × 35 mm^3^.

## 3. Results and Discussions

### 3.1. Chemical Features

**Figure 4:**
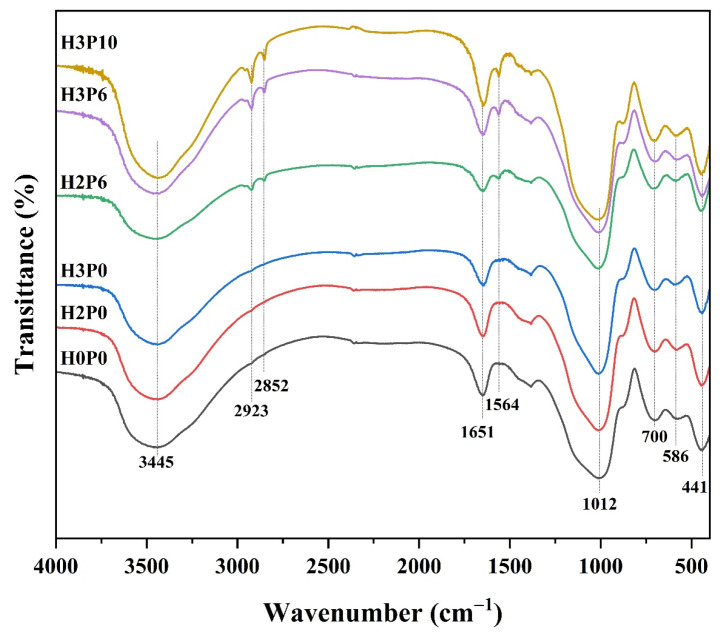
IR spectra of geopolymer foams with different H_2_O_2_ and palm olein content.

The infrared (IR) spectra of matrix and geopolymer foams with different H_2_O_2_ and palm olein content are presented in Figure 4.

For all samples, the absorption band around 1012 cm^−1^ corresponds to the symmetrical vibration of Si (Al)-O-Si bonds is observed, while the absorption peak corresponding to the in-plain bending vibration of Si-O bond appears at 441 cm^−1^ [39]. The absorption bands located at 700 and 586 cm^−1^ are related to the bending vibration of Si-O-Si and Si-O-Al bonds. This illustrates the formation of aluminosilicate structures in the matrix and geopolymer foams. Typical absorption bands corresponding to the stretching and bending vibration of H-O-H and O-H groups from free water and bound water are observed around 3445 and 1651 cm^−1^ [40], respectively. Three new absorption peaks around 2923, 2852, and 1564 cm^−1^ appear in the IR spectra of geopolymer foams with palm olein addition, corresponding to the stretching vibration of -CH_2_- and -COO^−^ [41]. This reveals the existence of long-chain fatty acid salts in geopolymer foams.

As shown in Figure 4, the IR spectra of geopolymer foams prepared with hydrogen peroxide only (H2P0 or H3P0) is similar to that of the matrix (H0P0), which indicates that the use of hydrogen peroxide has no significant effect on the chemical structure of geopolymer foams. However, fatty acid salts were found in geopolymer foams with palm olein addition, which were produced by the saponification between palm olein and alkali solution [42]. These fatty acid salts are highly effective surfactants and can have a significant impact on the formation process of geopolymer foams. Meanwhile, it can be found that the intensity of the characteristic absorption peaks of fatty acid salts grows with the increase in palm olein doping. This indicates that the added palm olein can adequately react with the alkali solution in the matrix.

**Figure 5:**
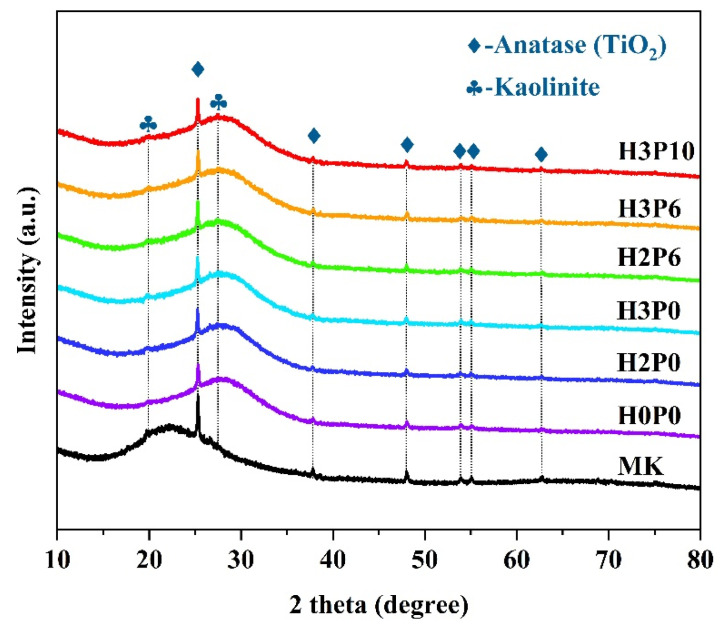
XRD patterns of matrix and geopolymer foams with different H_2_O_2_ and palm olein content.

Figure 5 shows the XRD patterns of matrix and geopolymer foams with different H_2_O_2_ and palm olein content. The diffraction peaks of all of the geopolymer foams are consistent with raw precursors. Some diffraction peaks of anatase (TiO_2_) and kaolinite are detected. The diffraction band located at 20–25° is attributed to the amorphous phase of MK [43,44]. After geopolymerization, a new diffraction hump in the range of 25–30° was detected in the matrix and geopolymer foams. In comparison with MK, a noticeable shift of the diffraction hump center was observed, which is consistent with the literature results [43,44,45,46]. The hump centered at ~28° can be attributed to the amorphous aluminosilicate gel, the typical binder phase in metakaolin-based geopolymer [43,45]. The existence of this amorphous hump coupled with the shift of the hump center demonstrated the occurrence of geopolymerization during geopolymer preparation. Typical characteristic peaks of amorphous structures were observed in all of the samples. This indicates that the use of H_2_O_2_ and palm olein does not significantly affect the alkali activation process of the precursor [40].

### 3.2. Morphology

**Figure 6:**
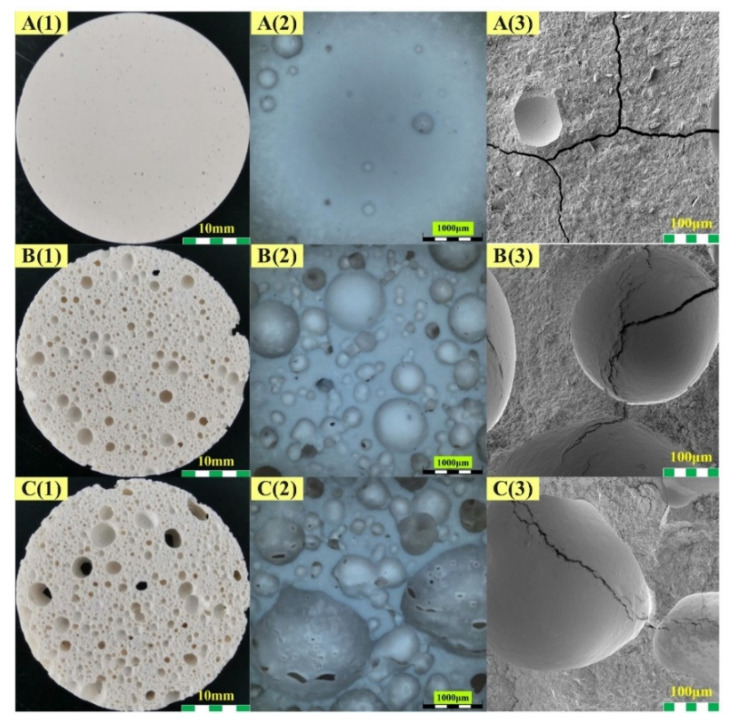
Morphology of matrix and geopolymer foams: (**A1**–**A3**) H0P0, (**B1**–**B3**) H2P0, (**C1**–**C3**) H3P0.

**Figure 7:**
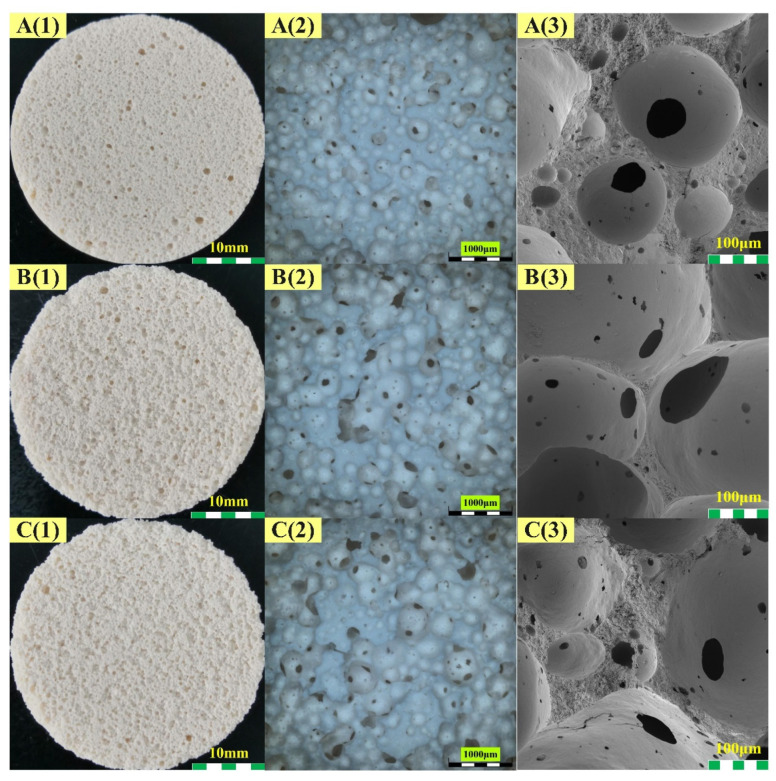
Morphology of geopolymer foams: (**A1**–**A3**) H2P6, (**B1**–**B3**) H3P6, (**C1**–**C3**) H3P10).

Figure 6 and Figure 7 present the optical and SEM images of the geopolymer matrix and geopolymer foams at different scales, of which (1) is the overall picture of each sample captured by the camera, (2) and (3) are the microtopographies obtained by optical-digital microscope and SEM, respectively.

With the addition of H_2_O_2_, a large number of pores were introduced into the matrix, resulting in a porous structure (Figure 6(B1,C1)). From the microscopic images, it can be noticed that these cells produced by the foaming agent are mainly closed cells. Comparing Figure 6(B2,C2), the average pore size of these samples increased significantly with the increase in H_2_O_2_ dosage. Meanwhile, many pores with a pore size of more than 350 μm were formed in these samples. The structural information of these pores could not be obtained by MIP.

Figure 7 illustrates the effect of palm oil on the morphology of the geopolymer foam prepared by the addition of H_2_O_2_. As can be seen from the optical images, the addition of palm oil resulted in a highly homogeneous pore structure in the geopolymer foams. The use of palm olein significantly influenced the pore morphology and pore size distribution of geopolymer foams. Microscopic images show that there is a considerable number of open pores with diameters of tens to hundreds of microns in the pore walls of the geopolymer foams with palm oil addition. The pore structure homogenization of the geopolymer foams is mainly caused by the saponification between palm oil and alkali activator. The surfactant, sodium fatty acid, generated in situ during the reaction was adsorbed on the gas-liquid interface created due to gas release. The foam walls were enhanced, and the surface tension of the mixture was reduced by the surfactant [3,32], which prevent drainage and coalescence of the foams from occurring.

By comparing Figure 6 and Figure 7, some cracks can be found in the matrix and in the geopolymer foams only containing H_2_O_2_, but not in those with palm olein addition. These cracks in the matrix and geopolymer foams shown in A3–C3 of Figure 6 are attributed to the high capillary pressures between dry and wet parts of the micropore network [47]. Due to the drop in ambient humidity, the geopolymer loses water rapidly, and thus cracks are initiated in the matrix [48]. With the increase in open porosity, the temperature and humidity in different parts of geopolymer foams can be equilibrated quickly, which effectively inhibits cracking. This is why no cracks are found on the microscopic images in Figure 7.

### 3.3. Pore size Distribution

**Figure 8:**
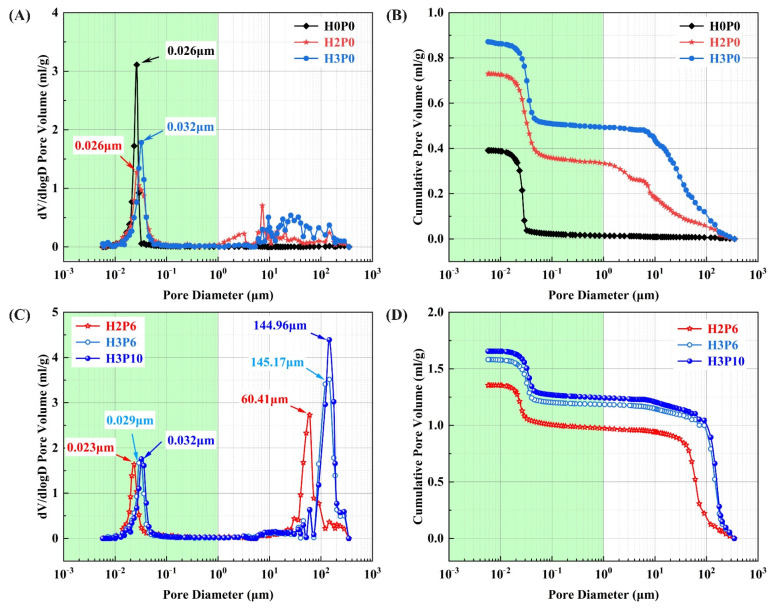
Pore size distribution of matrix and geopolymer foams: (**A**,**B**) H0P0, H2P0, and H3P0; (**C**,**D**) H2P6, H3P6, and H3P10.

MIP was employed to characterize pores in the 0.005–350 μm range in geopolymer foams. The pore size distribution of these pores cannot be precisely described by image measurements. Figure 8 presents the pore size distribution in geopolymer foams with different H_2_O_2_ and palm olein contents.

The pore size distribution data indicated that the geopolymer foams consist of two kinds of pores. The intrinsic pores derived from the geopolymerization ranged from 0.005 to 1 μm [49]. These macropores ranging from 1 to 350 μm are due to the decomposition of H_2_O_2_, since they do not present in the matrix.

The macropore distribution of geopolymer foams made with H_2_O_2_ only (H2P0 and H3P0) was scattered and irregular (Figure 8A). It is evident from the cumulative distribution curve that the volume of macropores increases with the growth of H_2_O_2_ dosage. For H2P0 and H3P0 samples, the volume of macropores increases from 0.333 mL/g to 0.492 mL/g (Figure 8B and Table 3, calculated from MIP data). The foaming agent also affects the structure of the intrinsic pores. For geopolymer foams with 3 wt.% H_2_O_2_ (H3P0), the intrinsic pore size was distributed between 0.01 and 0.05 μm, while for matrix, the size of the intrinsic pores was mainly between 0.01 and 0.03 μm. The expansion of the distribution range may result from the decomposition of H_2_O_2_ in the intrinsic pores. The critical pore entry diameter of H3P0 samples is 0.032 μm which is larger than 0.026 μm in the matrix, proving that H_2_O_2_ has the effect of increasing the size of the intrinsic pores in geopolymer foams.

For all of the geopolymer foams with palm olein addition, the pore size distribution was more regular than those without using the foam stabilizer. Both intrinsic pores and macropores show concentrated distribution. For macropores ranging from 1 to 350 μm, the critical pore entry diameter of H2P6 and H3P6 samples are 60.41 μm and 145.17 μm, respectively. It is revealed that the concentration of H_2_O_2_ dominates the critical pore entry diameter. In addition, the cumulative pore volume was significantly increased after the addition of palm olein. As shown in Table 3, for geopolymer foams with 2 and 3 wt.% H_2_O_2_, the cumulative pore volumes of macropores are 0.973 mL/g and 1.185 mL/g, which are three and two times higher than samples without palm olein (H2P0 and H3P0). The effect of palm olein content on macropores was also carried out. The macropores distribution is barely changed except for a slight increase in the peak, which means there will be an improvement in open porosity.

Another attractive finding is that the critical pore entry diameter of the intrinsic pores in geopolymer foams was decreased with palm olein addition, even smaller than that in the matrix. For H2P6 and H3P6 samples, the critical pore entry diameter in the range from 0.005 to 1 μm are 0.023 μm and 0.029 μm (Figure 8C), while for H2P0, H3P0, and the matrix (H0P0), the critical pore entry diameters are 0.026 μm, 0.029 μm, and 0.026 μm (Figure 8A), respectively.

This reduction in intrinsic pores was attributed to the sodium fatty acid generated in situ by saponification. Sodium fatty acid is an anionic surfactant with low critical micelle concentration (CMC). It can quickly form micelles at low concentrations. Sodium fatty acid molecules will be adsorbed on the pore surface to improve foam stability [42]. The intrinsic pore diameter will decrease when surfactant molecules generated in situ are adsorbed on the pore surface.

**Figure 9:**
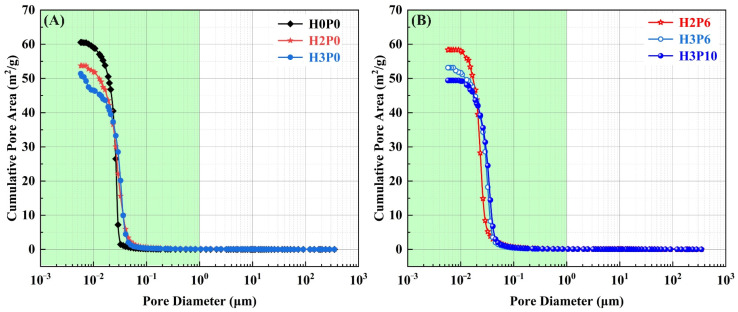
Cumulative pore area of matrix and geopolymer foams: (**A**) H0P0, H2P0, and H3P0; (**B**) H2P6, H3P6, and H3P10.

The pore size distribution and total pore volume changes will affect the total pore area, which is directly related to the adsorption capacity of geopolymer foams. The cumulative pore area data of matrix and geopolymer foams measured by MIP are presented in Figure 9. As shown in the curve, the total pore area of the samples is almost all derived from the intrinsic pores. These macropores have little to no effect on the cumulative pore area, though most of the pore volume was contributed by them. With 6 wt.% palm olein addition, a slight increase in the cumulative pore area of the H2P6 and H3P6 samples. It indicates that the reduction in intrinsic pores will be beneficial for improving the pore adsorption capacity.

However, when the palm olein content increased from 6 to 10 wt.% (H3P6 and H3P10), the total pore area of geopolymer foams with 3 wt.% H_2_O_2_ decreased from 53.158% to 49.421% (Figure 9 and Table 3). It can be seen from Figure 8C and Figure 9B that the difference in cumulative pore area is mainly derived from the pores ranging from 0.005 to 0.01 μm. These pores contributed a pore area of 5 m^2^/g in the H3P6 sample, while the H3P10 sample was almost free of pores in the same range. These pores disappeared in geopolymer foams with high palm olein content may be filled by surfactant micelles, so they cannot be detected by MIP.

### 3.4. Porosity

**Figure 10:**
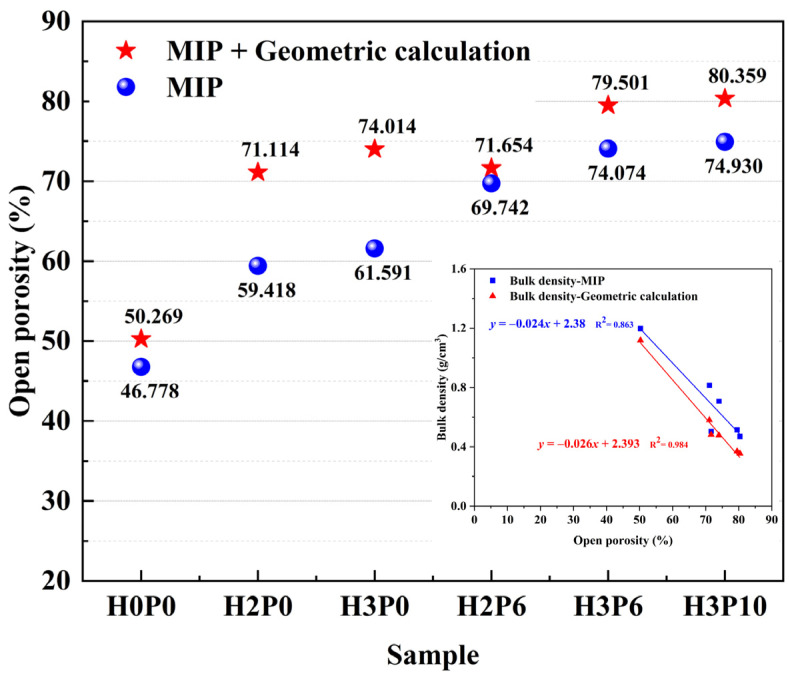
Open porosity of matrix and geopolymer foams.

The skeletal density and bulk density of samples obtained by MIP and geometric method are listed in Table 4. The open porosities of geopolymer foams calculated by Equation (1) are shown in Figure 10, along with the porosity determined by MIP.

The porosity of all of the geopolymer foams exceeded 70% with or without the addition of the foam stabilizer. For samples with 3 wt.% H_2_O_2_, the open porosity increased from 74.01% to 79.50%, using 6 wt.% palm olein. This proves that the use of palm olein can promote the opening of pores. However, for the sample with 2 wt.% H_2_O_2_ doping, there was only a slight increase in open porosity after using palm olein, which was inconsistent with the results measured by the MIP. It might be due to the fact that the use of palm olein improved the homogeneity of pores. Pores in these samples with diameters that exceed the upper limit of MIP cannot be detected. When adding the foam stabilizer, the pore size distribution of geopolymer foams was adjusted and concentrated so that most of the pores are less than 350 μm in size and can be detected. This also reveals that palm olein can optimize the pore size distribution and improve the open porosity in geopolymer foams. Comparing H3P6 and H3P10 samples, it can be found that increasing the amount of foam stabilizer can also slightly increase the open porosity. A linear regression analysis was employed to describe the effect of open porosity on the bulk density of geopolymer foams. Regression lines in Figure 10 showed a distinct negative correlation between the bulk density and open porosity of geopolymer foams, which is consistent with the finding in literature [5].

**Figure 11:**
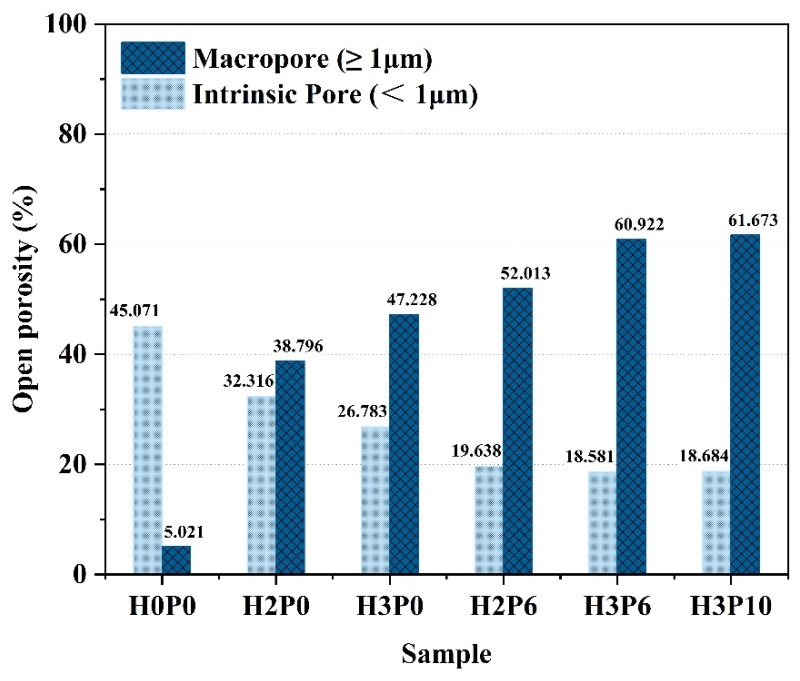
Graded open porosity of matrix and geopolymer foams.

More accurate macropore porosity can be obtained by combining MIP with geometric calculations. Table 5 presents the data extracted from the MIP and geometric calculations for the graded porosity analysis (Graded by diameter (*d*)). The graded porosity of macropores and intrinsic pores of the geopolymer foams is provided in Figure 11.

The porosity of the macropores was further improved using the foam stabilizer. For samples with H_2_O_2_ dosage of 2 and 3 wt.%, the porosity of the macropores increased from 38.80 and 47.23% to 52.01 and 60.92% with the addition of 6 wt.% palm oil, respectively. The intrinsic porosity in the same samples was also reduced accordingly. These results reveal the effect of palm oil on foam enhancement. The foam film was enhanced by the in situ generated surfactant, preventing drainage and consolidation from occurring. As a result, more of the gas generated by H_2_O_2_ can be trapped in the matrix and form pores. For geopolymer foams with H_2_O_2_ dosage, increasing the palm content from 6% to 10% has a negligible effect on the macroporosity.

### 3.5. Pore Shape Evaluation

**Figure 12:**
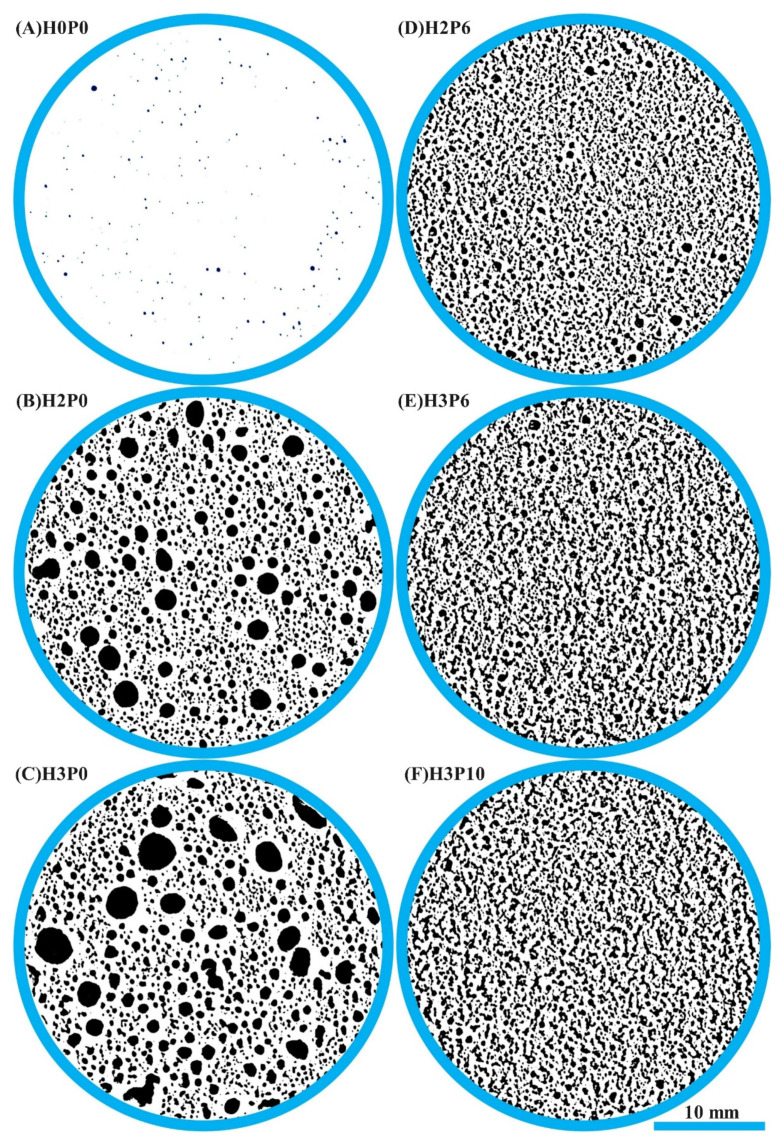
Cross-sectional binarized images of matrix and geopolymer foams.

Cross-sectional images of porous materials are often used for pore structural analysis, such as pore distribution frequency [24], porosity [50], and the shape of pores [4]. The cross-sectional binarized images of the matrix and geopolymer foams involved in this study are presented in Figure 12.

As can be seen in the binarized cross-sectional images, there are heteromorphic worm-like pore structures in the geopolymer foams in which palm olein was used. Observing the microscopic morphology of geopolymer foams, these heterogeneous macropores are composed of many uniform fine pores that have not fully merged. *Roundness* is a parameter used to assess the extent to which pores or particles deviate from a mathematically perfect circle. It can be used to evaluate the effect of palm olein on the shape of macropores in geopolymer foams [37,51].

**Figure 13:**
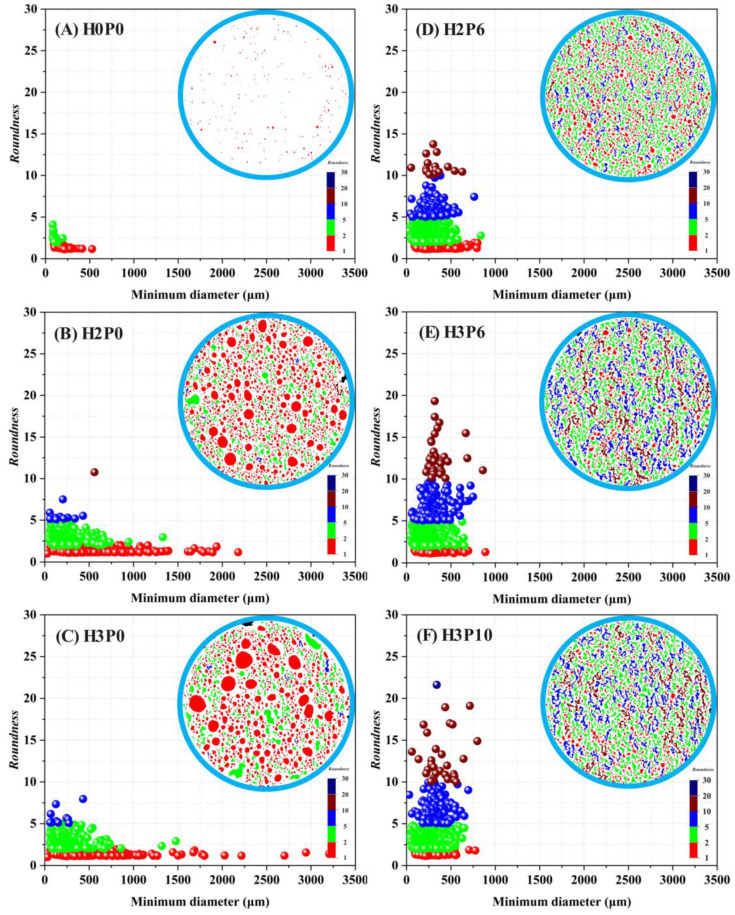
Cloud plots of pore roundness distribution of geopolymer foams.

The pore *roundness* can be deduced from Equation (1). The perimeter, area, and minimum diameter of each identifiable pore were captured by software for calculation and correlation analysis. Figure 13 gives a cloud plot of the *roundness* distribution of pores against the minimum diameters in geopolymer foams. A color cross-sectional view of the sample is provided in the upper right corner of each image, where each pore was filled using the color corresponding to the *roundness*.

**Figure 14:**
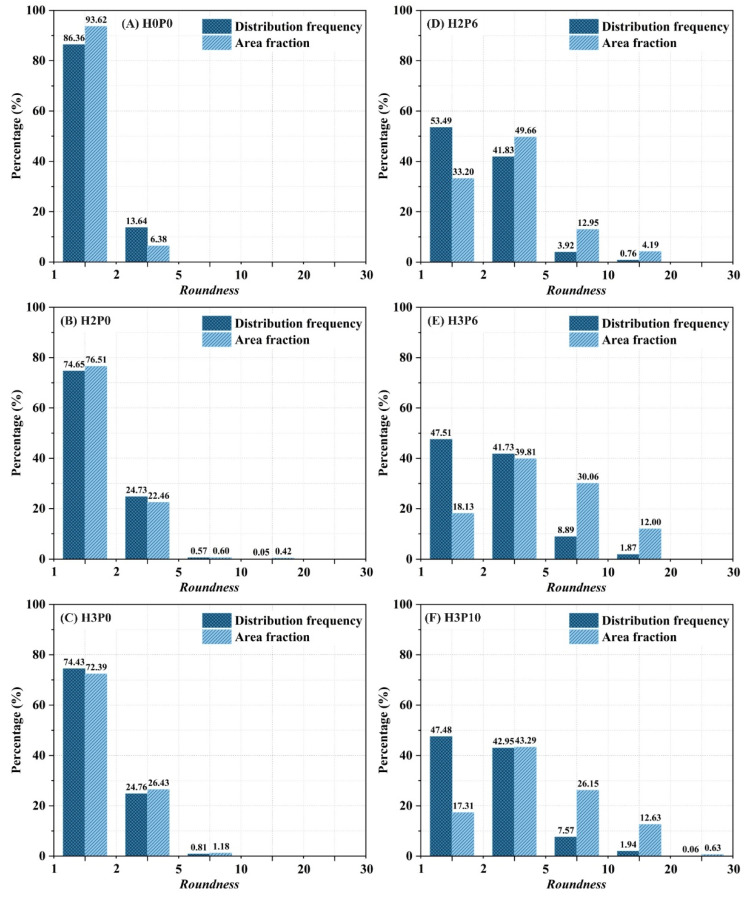
Pore distribution frequency and area fraction of geopolymer foams.

As shown in Figure 13B,C, in the samples using only H_2_O_2_, the *roundness* of almost all of the pores was below 5. The *roundness* of pores with a diameter greater than 1000 μm is practically located in the range from 1 to 2. The cloud map of pore distribution shows that the shape of pores with a *roundness* of less than 2 is mostly circular or sub-circular. It means that these pores are isolated and not interconnected to others. The distribution frequency and area fraction of pores with different *roundness* are illustrated in Figure 14.

The trend of the pore *roundness* distribution is the same as in Figure 13. For the H2P0 and H3P0 samples, about three-fourths of the pores have *roundness* between 1 and 2. It can also be found that the area fraction is consistent with the distribution frequency of pores, which indicates that the *roundness* distribution has similar regularity in all pore size ranges. The macropores in geopolymer foams using H_2_O_2_ only are nearly closed pores with a low interconnectivity, which is consistent with the presented microscopic morphology in Figure 6.

Figure 13D–F shows that many pores with *roundness* higher than 5 were found in the geopolymer foams containing palm olein. These pores correspond to the heterogeneous pores on the color cloud map. Moreover, the shape of these pores becomes more irregular as the *roundness* increases. As presented in Figure 14E, for H2P6 samples, the proportion of pores with *roundness* between 5 and 10 is 3.92%, which is 7 times as high as that of geopolymer foams with H_2_O_2_ only. In parallel, the proportion of pores with a *roundness* of less than 2 has significantly decreased. In the H3P10 sample, pores with *roundness* greater than 20 were observed. In addition, the pore diameter distribution in geopolymer foams was significantly narrowed after using palm olein, as shown in Figure 13. As with the H3P6 sample, the pore diameter distribution ranges from 0 to 1000 μm, one-third of the geopolymer foams without palm olein addition.

It can be observed from Figure 14 that pores with large *roundness* (larger than 5) contribute more to the total area, despite their low frequency of distribution. This indicates that the macropores with larger *roundness* are composed of more individual fine pores that are not fully integrated. In other words, the *roundness* of a heteromorphic pore is positively related to the number of individual pores it contains.

### 3.6. Connectivity of Pores

As shown by binarized and microscopic images, the geopolymer foams with the addition of palm olein contained many heteromorphic worm-like pores. These irregular pores, formed by interconnected individual fine pores, significantly affect the macropore connectivity of geopolymer foams. As mentioned in the previous section, the *roundness* of these pores is correlated positively with the number of individual pores. This makes it possible to use *roundness* to evaluate the local connectivity of these irregular pores.

**Figure 15:**
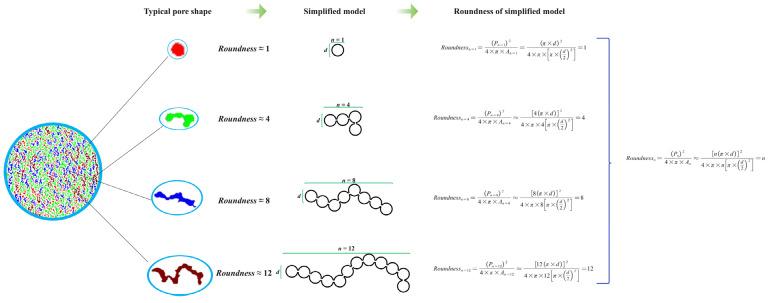
A simplified interconnected pore models of geopolymer foams.

Here, we define the local connectivity (*κ_Local_*) as the number (*n*) of internally connected pores that constitute an irregular pore. A simplified interconnected pore model was proposed to derive the relationship between each irregular pore’s *roundness* and local connectivity, as shown in Figure 15. The model is based on 2 assumptions: (1) all individual pores are identical spheres; (2) except for the pores located at the ends, the remaining pores are only connected to other two pores through single points. It can be summarized that the model is only related to the number of pores involved and is applicable to various shapes of pores that satisfy the assumptions.

As shown in Figure 15, for an irregular pore consisting of *n* internally connected pores, the calculated pore *roundness* is also approximately equal to *n*. According to the definition of local connectivity in this study, the local connectivity of the corresponding irregular pore is *n*. It means that the pore *roundness* can be used to evaluate the local connectivity quantitatively. Based on a simplified linear connectivity model, a perfect linear relationship exists between pore *roundness* and local connectivity.

The local connectivity of all of the pores determines the global connectivity of geopolymer foams. As shown in Figure 13, the connectivity in geopolymer foams with different H_2_O_2_ and palm olein contents is significantly different.

Standard deviation is often used to evaluate the degree of dispersion of a data set. Here, an attempt to evaluate the collated connectivity of geopolymer foams was processed by calculating the standard deviation of the local connectivity of all pores in cross-sectional images.

In this study, the global connectivity (*κ_Global_*) of geopolymer foams was defined as the standard deviation (σ (*κ*)) of the *roundness* of all identifiable pores on the two-dimensional images. It can be calculated by Equation (6):

(6) $$\kappa_{Global}=\sigma \;\left(\kappa \right)=\sqrt{\frac{\sum_{i=1}^{n}\;{\left(\kappa_{i}-\kappa_{avg}\right)}^{2}}{n}}$$

where *κ_i_* is the local connectivity of the *i*th pores, *κ_avg_* is the average of the local connectivity of all pores, and *n* is the total number of pores.

The global connectivity and the total number of pores for statistical analysis are listed in Table 6. The global connectivity of geopolymer foams showed a significant increase after using palm olein. For samples H2P6 and H3P6, the global connectivity is twice and three times higher than these without palm olein. It indicates that palm olein can improve the pore connectivity of geopolymer foams with the cooperation of H_2_O_2_. However, the global connectivity of geopolymer foams was not significantly improved by increasing the amount of palm olein with the same H_2_O_2_ dosage. The result suggests there is an optimal doping of palm olein for a defined geopolymer foams system and that overdosing does not further affect the pore connectivity of geopolymer foams.

**Figure 16:**
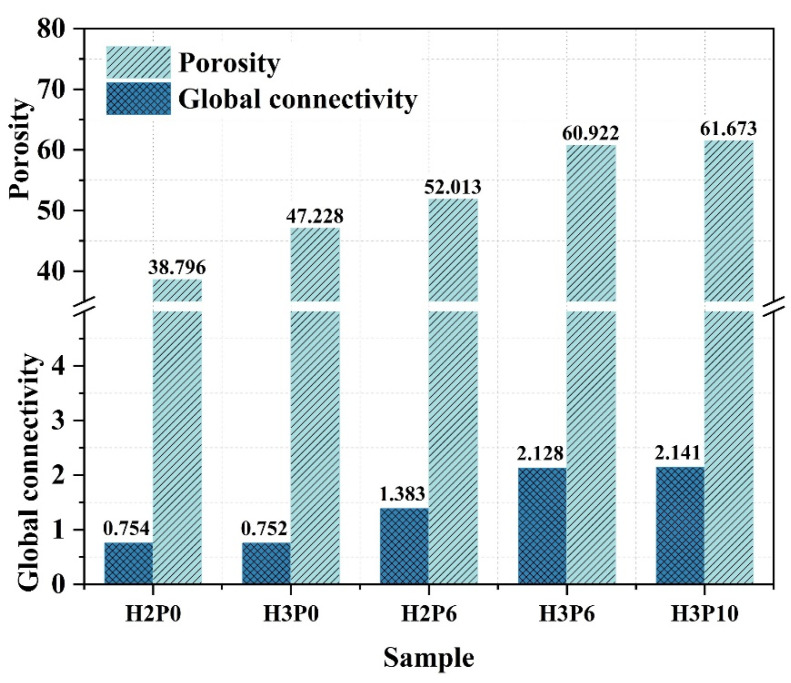
Global connectivity and macropore porosity of geopolymer foams.

**Figure 17:**
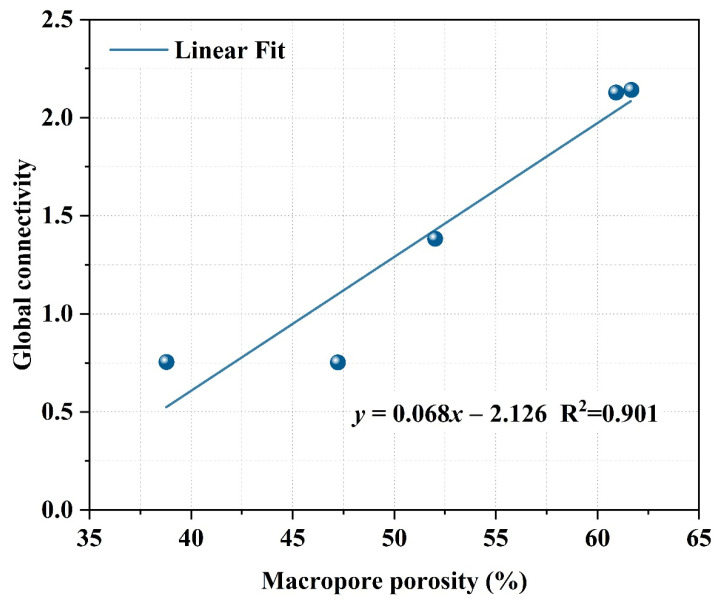
Regression analysis of pore connectivity of geopolymer foams.

The open porosity of porous materials is often used to describe the pore connectivity in porous structures qualitatively. The histograms of connectivity and macropore porosity for different samples shown in Figure 16 illustrate a positive correlation between these two parameters. A simple linear regression analysis was performed to verify the validity of this quantitative connectivity evaluation method. The regression line, fitting equation, and R-squared value presented in Figure 17 reveal that the values derived by this method can represent the connectivity of the pores in geopolymer foams.

However, the simplified model was built based on the two assumptions previously mentioned. For geopolymer foams with a wider pore size distribution and more complex connectivity, the model needs to be modified accordingly.

### 3.7. Water Absorption

**Figure 18:**
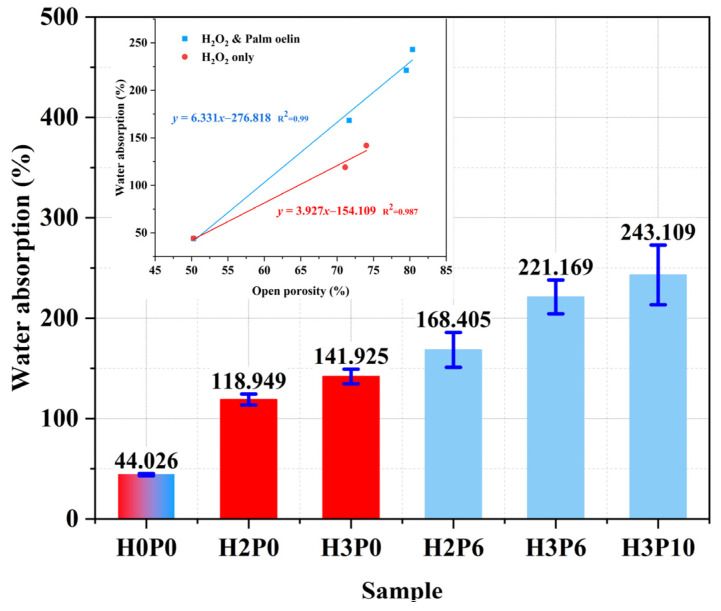
Water absorption of matrix and geopolymer foams.

Figure 18 shows the water absorption of the geopolymer foams with different H_2_O_2_ and palm oil contents. It can be seen that the water absorption of the geopolymer foams increases significantly with the amount of H_2_O_2_. The water absorption of the sample with the addition of 3 wt.% H_2_O_2_ was 141.925%, which was 2 times higher than that of the matrix. The water absorption of sample H3P6 has reached 221.169%. This indicates that the use of palm olein can further improve the absorption capacity of geopolymer foams. A similar trend was observed in the samples with low H_2_O_2_ incorporation.

The water absorption of geopolymer foams is mainly influenced by the open pore structure. The effects of open porosity on the water absorption of geopolymer foams with or without palm olein addition were discussed and presented in the accompanying figure of Figure 18. It is evident that the open porosity of geopolymer foams and their water absorption is significantly positively correlated regardless of the addition of palm olein, which means that the water absorption of geopolymer foams can be improved by increasing the open porosity. It is worth mentioning that the regression line slope was higher for geopolymer foams with palm olein addition compared to those with H_2_O_2_ only. This indicates that the former has better water absorption, which also validates the positive effect of palm olein on increasing the open porosity of geopolymer foams. In summary, geopolymer foams with higher open porosity have better water absorption capacity. This makes geopolymer foams have the potential to be used in moisture conditioning and composite carriers.

### 3.8. Thermal Conductivity

**Figure 19:**
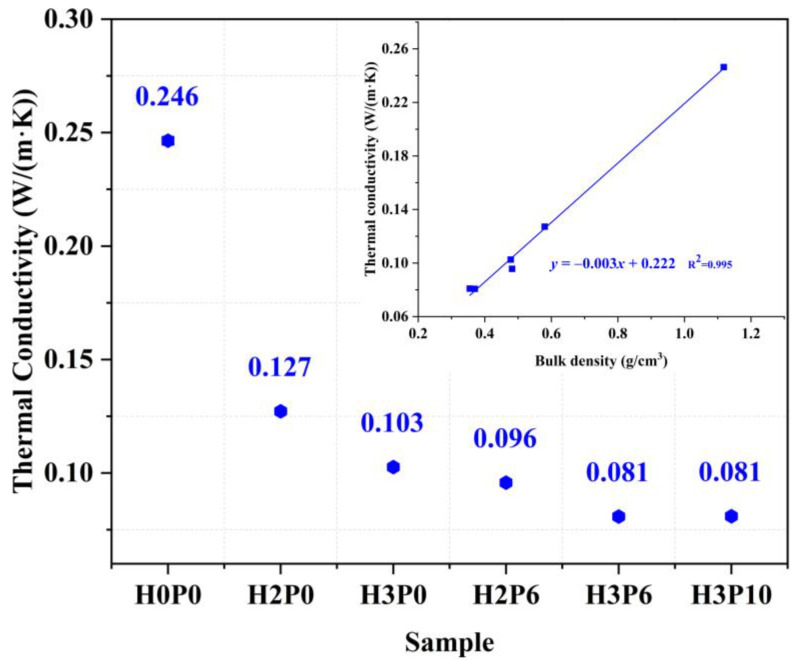
Thermal conductivity of matrix and geopolymer foams.

The effect of H_2_O_2_ and palm olein doping on the thermal conductivity of geopolymer foams is shown in Figure 19. It can be found that the thermal conductivity of samples gradually decreases with the increase in H_2_O_2_ dosages. As the H_2_O_2_ content increased from 2 to 3 wt.%, the thermal conductivity of geopolymer foams reduced from 0.127 to 0.103 W/ (m·K), which is only two-fifths of that for the matrix. This is because the foaming agent introduces plenty of closed pores into the matrix [37].

With the use of palm olein, geopolymer foams with lower thermal conductivity were obtained. For the H3P6 sample, thermal conductivity as low as 0.081 W/(m·K) was measured. This indicates that the thermal insulation properties of geopolymer foams can be improved with the addition of palm olein. The thermal conductivity of geopolymer foams did not change obviously by further increasing the amount of palm olein, which is attributed to the fact that excessive palm olein dosage can no longer significantly affect the pore structure. It can be concluded that geopolymer foams with different thermal insulation properties can be obtained by adjusting the amounts of H_2_O_2_ and palm olein. Regression analysis showed that the thermal conductivity of geopolymer foams was highly linear correlated with their bulk density (R^2^ = 0.995). Combining the results presented in Figure 10, we can infer that there is also a negative correlation between open porosity and thermal conductivity in geopolymer foams.

### 3.9. Compressive Strength

**Figure 20:**
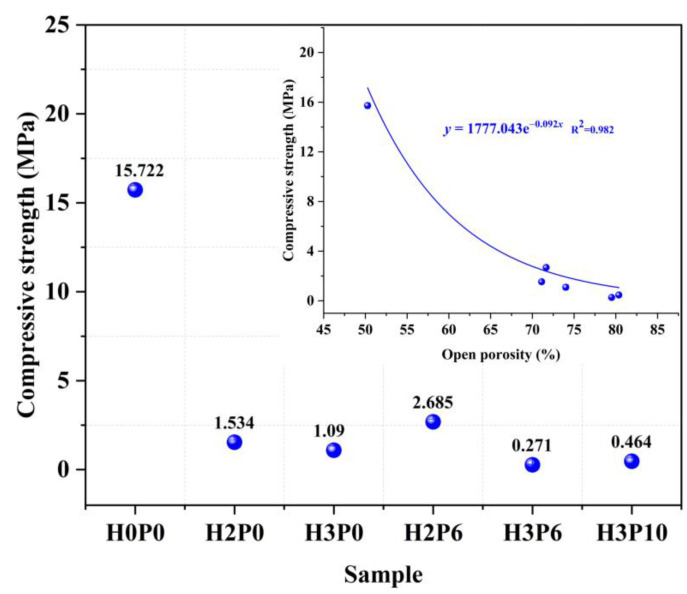
Compressive strength of matrix and geopolymer foams.

The effects of H_2_O_2_ and palm olein on the compressive strength of geopolymer foams are presented in Figure 20. With H_2_O_2_ content increasing, the compressive strength of geopolymer foams declined regardless of whether palm olein was added. However, palm olein positively affects the strength development of geopolymer foams with low H_2_O_2_ doping. For samples with 2 wt.% H_2_O_2_, the compressive strength increased by 75% from 1.534 to 2.685 MPa after adding 6 wt.% palm olein. This enhancement effect should be attributed to the modulation of pore distribution in geopolymer foams by palm olein. As shown in Figure 8, the H2P6 sample possessed a more homogeneous pore structure than H2P0, which is beneficial for compressive strength.

All of the regression analyses indicate that the various properties of geopolymer foams are closely related to the pore characteristics. As mentioned before, the open porosity significantly affects the water absorption, mechanical behaviors, and thermal conductivity of geopolymer foams. This study confirms the positive role of palm olein in the optimization and regulation of pore structure. With the synergistic effect of H_2_O_2_ and palm olein, geopolymer foams featuring lightweight, low thermal conductivity, and good mechanical properties can be prepared. Compared with lightweight geopolymers reported in the literature [6,46,52], the geopolymer foams fabricated in this study (H2P6) possess comparable low thermal conductivity, allowing them to be used for building thermal insulation. Moreover, geopolymer foams with high open porosity show favorable capabilities in heavy metal adsorption [53,54], liquid phase separation [55], and wastewater purification [56]. Therefore, the geopolymer foams prepared by this research route have significant potential in all the fields mentioned above.

Many pieces of literature reported an exponential correlation between porosity and compressive strength of geopolymer foams [4,5]. In this study, a non-linear regression analysis was performed to discuss the relations between these two parameters of geopolymer foams. The regression curve and correlation coefficient in Figure 20 illustrate an exponential correlation between the compressive strength of geopolymer foams and their open porosity, which means that geopolymer foams with adequate mechanical properties can be obtained by modulating the porosity.

## 4. Conclusions

Geopolymer foams with high porosity and hierarchical pore structure were prepared using the direct foaming method with H_2_O_2_ as the foaming agent and palm olein as the foaming stabilizer. The chemical features and pore structure of geopolymer foams with and without palm olein addition were investigated by experiments and calculations. Meanwhile, the water absorption, thermal insulation, and mechanical properties of geopolymer foams were evaluated. The main findings obtained in this study are as follows.

Fatty acid salt surfactant was generated in situ in the geopolymer foams after adding palm olein. However, the addition of H_2_O_2_ and palm olein did not affect the phase composition of the amorphous structure in the geopolymer matrix.

The macropores in geopolymer foams introduced by H_2_O_2_ were regulated by the surfactant generated in situ via saponification of palm olein with alkali solution. A uniformly concentrated macropore distribution was obtained in geopolymer foams using palm olein. The novel foam stabilizer also affects the structure of the intrinsic pores formed by geopolymerization. With palm olein addition, the intrinsic pores in geopolymer foams were fine-tuned, which resulted in an elevated total pore area.

The addition of palm olein has the effect of increasing the open porosity of the geopolymer foams. However, the impacts on macropores and intrinsic pores are different. The open porosity of macropores in geopolymer foams significantly increases after palm olein use, while the opposite effect was observed for intrinsic pores.

The roundness analysis indicates that irregular worm-like pores were formed in geopolymer foams with palm olein addition. These irregular pores are composed of interconnected, not fully consolidated individual fine pores. A simplified interconnected pore model based on the statistical analysis of pore roundness was proposed to quantify the macropore connectivity of geopolymer foams. The results demonstrated that the macropore connectivity of geopolymer foams was significantly improved using palm olein. The validity of the connectivity evaluation method based on the model was verified by correlation analysis.

The water absorption and thermal insulation capacity of geopolymer foams were significantly affected by the porosity and pore connectivity due to the addition of H_2_O_2_ and palm olein. By adding 3 wt.% H_2_O_2_ and 6 wt.% palm olein, geopolymer foams with water absorption of 221.169% and thermal conductivity of 0.081 W/(m·K) can be obtained. The compressive strength of geopolymer foams reduces with H_2_O_2_ content increasing, while palm olein positively affects the strength development of geopolymer foams with low H_2_O_2_ doping. Regression analysis showed strong correlations between the above properties and the pore characteristics of geopolymer foams.

From the results of this study, it can be concluded that palm olein is an efficient foam stabilizer that can be used to prepare eco-friendly geopolymer foams with different pore structures. However, the mechanism of pore structure evolution of geopolymer foams fabricated using palm olein still needs to be systematically investigated. Through further modification of the pore structure, geopolymer foams can be used in a wide range of applications such as adsorption, filtration, and reaction carriers.

## Figures and Tables

**Table 1:** Chemical composition of metakaolin (wt.%).

Oxide	SiO_2_	Al_2_O_3_	TiO_2_	Fe_2_O_3_	Na_2_O	Cr_2_O_3_	L.O.I. *
MK	53.15	42.28	3.58	0.56	0.39	0.03	0.08

* L.O.I.: Loss of ignition at 950 °C.

**Table 2:** Mix proportions of the samples (g).

Type	Sample	Metakaolin	Sodium Silicate	NaOH	Water	H_2_O_2_	Palm Olein
Matrix	H0P0	100.0	100.73	15.33	54.61	0	0
Geopolymer foams	H2P0	100.0	100.73	15.33	54.61	2	0
	H3P0	100.0	100.73	15.33	54.61	3	0
	H2P6	100.0	100.73	15.33	54.61	2	6
	H3P6	100.0	100.73	15.33	54.61	3	6
	H3P10	100.0	100.73	15.33	54.61	3	10

**Table 3:** Pore volume, pore area, and average pore diameter of geopolymer foams from MIP.

Sample	Pore Volume 0.005–1 μm (mL/g)	Pore Volume 1–350 μm (mL/g)	Total Pore Volume (mL/g)	Total Pore Area (m^2^/g)	Average Pore Diameter (nm)
H0P0	0.377	0.014	0.391	60.563	25.824
H2P0	0.397	0.333	0.729	53.731	54.300
H3P0	0.379	0.492	0.871	51.420	67.771
H2P6	0.381	0.973	1.354	58.370	92.815
H3P6	0.397	1.185	1.581	53.158	118.981
H3P10	0.413	1.242	1.655	49.421	133.951

**Table 4:** Skeletal and bulk densities of geopolymer foams.

Sample	Skeletal Density by MIP (g/cm^3^)	Bulk Density at 0.52 Psia by MIP (g/cm^3^)	Bulk Density by Geometric Calculation (g/cm^3^)
H0P0	2.248	1.197	1.118 ± 0.010
H2P0	2.007	0.815	0.580 ± 0.006
H3P0	1.841	0.707	0.478 ± 0.024
H2P6	1.726	0.504	0.482 ± 0.002
H3P6	1.702	0.515	0.370 ± 0.002
H3P10	1.807	0.469	0.355 ± 0.001

**Table 5:** Graded open porosity of geopolymer foams.

Sample	Open Porosity (%)			
	*d* < 1 μm	1 μm ≤ *d* < 350 μm	*d* ≥ 350 μm	*d* ≥ 1 μm
H0P0	45.071	1.709	3.492	5.201
H2P0	32.316	27.102	11.694	38.796
H3P0	26.783	34.808	12.420	47.228
H2P6	19.638	50.103	1.910	52.013
H3P6	18.581	55.493	5.429	60.922
H3P10	18.684	56.246	5.427	61.673

**Table 6:** Global connectivity and the total number of pores of geopolymer foams.

Sample	*n*	*κ_Global_*
H2P0	1929	0.754
H3P0	1357	0.752
H2P6	2247	1.383
H3P6	1608	2.128
H3P10	1546	2.141

## Data Availability

Not applicable.

## References

[1] Hassan A., Arif M., Shariq M. (2019). Use of Geopolymer Concrete for a Cleaner and Sustainable Environment—A Review of Mechanical Properties and Microstructure. J. Clean. Prod..

[2] Duan P., Yan C., Zhou W., Luo W., Shen C. (2015). An Investigation of the Microstructure and Durability of a Fluidized Bed Fly Ash-Metakaolin Geopolymer after Heat and Acid Exposure. Mater. Des..

[3] Dhasindrakrishna K., Pasupathy K., Ramakrishnan S., Sanjayan J. (2021). Progress, Current Thinking and Challenges in Geopolymer Foam Concrete Technology. Cem. Concr. Compos..

[4] Zhang Z., Wang H. (2016). The Pore Characteristics of Geopolymer Foam Concrete and Their Impact on the Compressive Strength and Modulus. Front. Mater..

[5] Sornlar W., Wannagon A., Supothina S. (2021). Stabilized Homogeneous Porous Structure and Pore Type Effects on the Properties of Lightweight Kaolinite-Based Geopolymers. J. Build. Eng..

[6] Jaya N.A., Yun-Ming L., Cheng-Yong H., Abdullah M.M.A.B., Hussin K. (2020). Correlation between Pore Structure, Compressive Strength and Thermal Conductivity of Porous Metakaolin Geopolymer. Constr. Build. Mater..

[7] Liu M.Y.J., Alengaram U.J., Jumaat M.Z., Mo K.H. (2014). Evaluation of Thermal Conductivity, Mechanical and Transport Properties of Lightweight Aggregate Foamed Geopolymer Concrete. Energy Build..

[8] Krzywoń R., Dawczyński S. (2021). Strength Parameters of Foamed Geopolymer Reinforced with Gfrp Mesh. Materials.

[9] Gao H., Liao L., Liu H., Mei L., Wang Z., Huang D., Lv G., Zhu G., Wang C. (2020). Optimization of Thermal Insulation Performance of Porous Geopolymers under the Guidance of Thermal Conductivity Calculation. Ceram. Int..

[10] Peng X., Shuai Q., Li H., Ding Q., Gu Y., Cheng C., Xu Z. (2020). Fabrication and Fireproofing Performance of the Coal Fly Ash-Metakaolin-Based Geopolymer Foams. Materials.

[11] Peng X., Li H., Shuai Q., Wang L. (2020). Fire Resistance of Alkali Activated Geopolymer Foams Produced from Metakaolin and Na_2_O_2_. Materials.

[12] Gao H., Liu H., Liao L., Mei L., Zhang F., Zhang L., Li S., Lv G. (2020). A Bifunctional Hierarchical Porous Kaolinite Geopolymer with Good Performance in Thermal and Sound Insulation. Constr. Build. Mater..

[13] Liu X., Hu C., Chu L. (2020). Microstructure, Compressive Strength and Sound Insulation Property of Fly Ash-Based Geopolymeric Foams with Silica Fume as Foaming Agent. Materials.

[14] Ge Y., Cui X., Kong Y., Li Z., He Y., Zhou Q. (2015). Porous Geopolymeric Spheres for Removal of Cu(II) from Aqueous Solution: Synthesis and Evaluation. J. Hazard. Mater..

[15] Humberto Tommasini Vieira Ramos F.J., Vieira Marques M.d.F., de Oliveira Aguiar V., Jorge F.E. (2022). Performance of Geopolymer Foams of Blast Furnace Slag Covered with Poly(Lactic Acid) for Wastewater Treatment. Ceram. Int..

[16] Roviello G., Chianese E., Ferone C., Ricciotti L., Roviello V., Cioff R., Tarallo O. (2019). Hybrid Geopolymeric Foams for the Removal of Metallic Ions from Aqueous Waste Solutions. Materials.

[17] Reeb C., Pierlot C., Davy C., Lambertin D. (2021). Incorporation of Organic Liquids into Geopolymer Materials—A Review of Processing, Properties and Applications. Ceram. Int..

[18] Gasca-Tirado J.R., Manzano-Ramírez A., Velázquez-Castillo R.R., Gómez-Luna B.E., Nava-Mendoza R.F., López-Romero J.M., Apátiga-Castro L.M., Rivera-Muñoz E.M. (2019). Porous Geopolymer as a Possible Template for a Phase Change Material. Mater. Chem. Phys..

[19] Hassan A., Rashid Y., Mourad A.H.I., Ismail N., Laghari M.S. (2019). Thermal and Structural Characterization of Geopolymer-Coated Polyurethane Foam-Phase Change Material Capsules/Geopolymer Concrete Composites. Materials.

[20] Bai C., Colombo P. (2018). Processing, Properties and Applications of Highly Porous Geopolymers: A Review. Ceram. Int..

[21] Zhang Z., Provis J.L., Reid A., Wang H. (2014). Geopolymer Foam Concrete: An Emerging Material for Sustainable Construction. Constr. Build. Mater..

[22] Hajimohammadi A., Ngo T., Mendis P. (2017). How Does Aluminium Foaming Agent Impact the Geopolymer Formation Mechanism?. Cem. Concr. Compos..

[23] Anggarini U., Pratapa S., Purnomo V., Sukmana N.C. (2019). A Comparative Study of the Utilization of Synthetic Foaming Agent and Aluminum Powder as Pore-Forming Agents in Lightweight Geopolymer Synthesis. Open Chem..

[24] Kioupis D., Zisimopoulou A., Tsivilis S., Kakali G. (2021). Development of Porous Geopolymers Foamed by Aluminum and Zinc Powders. Ceram. Int..

[25] Prud’homme E., Michaud P., Joussein E., Peyratout C., Smith A., Arrii-Clacens S., Clacens J.M., Rossignol S. (2010). Silica Fume as Porogent Agent in Geo-Materials at Low Temperature. J. Eur. Ceram. Soc..

[26] Hajimohammadi A., Ngo T., Mendis P., Nguyen T., Kashani A., van Deventer J.S.J. (2017). Pore Characteristics in One-Part Mix Geopolymers Foamed by H_2_O_2_: The Impact of Mix Design. Mater. Des..

[27] Cilla M.S., de Mello Innocentini M.D., Morelli M.R., Colombo P. (2017). Geopolymer Foams Obtained by the Saponification/Peroxide/Gelcasting Combined Route Using Different Soap Foam Precursors. J. Am. Ceram. Soc..

[28] Ji Z., Su L., Pei Y. (2021). Characterization and Adsorption Performance of Waste-Based Porous Open-Cell Geopolymer with One-Pot Preparation. Ceram. Int..

[29] Hajimohammadi A., Ngo T., Mendis P. (2018). Enhancing the Strength of Pre-Made Foams for Foam Concrete Applications. Cem. Concr. Compos..

[30] Strozi Cilla M., Colombo P., Raymundo Morelli M. (2014). Geopolymer Foams by Gelcasting. Ceram. Int..

[31] Bai C., Ni T., Wang Q., Li H., Colombo P. (2018). Porosity, Mechanical and Insulating Properties of Geopolymer Foams Using Vegetable Oil as the Stabilizing Agent. J. Eur. Ceram. Soc..

[32] Bai C., Colombo P. (2017). High-Porosity Geopolymer Membrane Supports by Peroxide Route with the Addition of Egg White as Surfactant. Ceram. Int..

[33] Zhang X., Bai C., Qiao Y., Wang X., Jia D., Li H., Colombo P. (2021). Porous Geopolymer Composites: A Review. Compos. Part A Appl. S..

[34] Patterson H.B.W. (2011). Hydrogenation of Fats and Oils: Theory and Practice.

[35] Hlaváček P., Šmilauer V., Škvára F., Kopecký L., Šulc R. (2015). Inorganic Foams Made from Alkali-Activated Fly Ash: Mechanical, Chemical and Physical Properties. J. Eur. Ceram. Soc..

[36] Papa E., Medri V., Kpogbemabou D., Morinière V., Laumonier J., Vaccari A., Rossignol S. (2016). Porosity and Insulating Properties of Silica-Fume Based Foams. Energy Build..

[37] Xu F., Gu G., Zhang W., Wang H., Huang X., Zhu J. (2018). Pore Structure Analysis and Properties Evaluations of Fly Ash-Based Geopolymer Foams by Chemical Foaming Method. Ceram. Int..

[38] Saito Y., Matsuo S., Kanai T., Toishi A., Uchida A., Yamazaki Y., Matsushita Y., Aoki H., Nomura S., Hayashizaki H. (2014). Effect of Random Pore Shape, Arrangement and Nonadhesion Grain Boundaries on Coke Strength. ISIJ Int..

[39] Chen X., Niu Z., Wang J., Zhu G.R., Zhou M. (2018). Effect of Sodium Polyacrylate on Mechanical Properties and Microstructure of Metakaolin-Based Geopolymer with Different SiO_2_/Al_2_O_3_ Ratio. Ceram. Int..

[40] Yan S., Feng X., Yang Y., Xing P. (2021). Effects of High-Temperature Exposure on Properties of Lightweight Geopolymer Foams Incorporating Diatomite Powders. Int. J. Appl. Ceram. Technol..

[41] Rohman A., Man Y.C. (2011). Determination of Sodium Fatty Acid in Soap Formulation Using Fourier Transform Infrared (FTIR) Spectroscopy and Multivariate Calibrations. J. Surfactants Deterg..

[42] Cilla M.S., Morelli M.R., Colombo P. (2014). Open Cell Geopolymer Foams by a Novel Saponification/Peroxide/Gelcasting Combined Route. J. Eur. Ceram. Soc..

[43] Qiao Y., Li X., Bai C., Li H., Yan J., Wang Y., Wang X., Zhang X., Zheng T., Colombo P. (2021). Effects of Surfactants/Stabilizing Agents on the Microstructure and Properties of Porous Geopolymers by Direct Foaming. J. Asian. Ceram. Soc..

[44] Chen L., Wang Z., Wang Y., Feng J. (2016). Preparation and Properties of Alkali Activated Metakaolin-Based Geopolymer. Materials.

[45] Rahier H., van Mele B., Biesemans M., Wastiels J., Wu X. (1996). Low-Temperature Synthesized Aluminosilicate Glasses. J. Mater. Sci..

[46] Novais R.M., Ascensão G., Buruberri L.H., Senff L., Labrincha J.A. (2016). Influence of Blowing Agent on the Fresh- and Hardened-State Properties of Lightweight Geopolymers. Mater. Des..

[47] Kuenzel C., Vandeperre L.J., Donatello S., Boccaccini A.R., Cheeseman C. (2012). Ambient Temperature Drying Shrinkage and Cracking in Metakaolin-Based Geopolymers. J. Am. Ceram.Soc..

[48] Perera D.S., Uchida O., Vance E.R., Finnie K.S. (2007). Influence of Curing Schedule on the Integrity of Geopolymers. J. Mater. Sci..

[49] Papa E., Medri V., Benito P., Vaccari A., Bugani S., Jaroszewicz J., Swieszkowski W., Landi E. (2015). Synthesis of Porous Hierarchical Geopolymer Monoliths by Ice-Templating. Micropor. Mesopor. Mater..

[50] Kioupis D., Kavakakis C., Tsivilis S., Kakali G. (2018). Synthesis and Characterization of Porous Fly Ash-Based Geopolymers Using Si as Foaming Agent. Adv. Mater. Sci. Eng..

[51] Gu G., Xu F., Ruan S., Huang X., Zhu J., Peng C. (2020). Influence of Precast Foam on the Pore Structure and Properties of Fly Ash-Based Geopolymer Foams. Constr. Build. Mater..

[52] Palmero P., Formia A., Antonaci P., Brini S., Tulliani J.-M. (2015). Geopolymer Technology for Application-Oriented Dense and Lightened Materials. Elaboration and Characterization. Ceram. Int..

[53] He P.Y., Zhang Y.J., Chen H., Han Z.C., Liu L.C. (2020). Low-Cost and Facile Synthesis of Geopolymer-Zeolite Composite Membrane for Chromium(VI) Separation from Aqueous Solution. J. Hazard. Mater..

[54] Yan C., Guo L., Ren D., Duan P. (2019). Novel Composites Based on Geopolymer for Removal of Pb(II). Mater. Lett..

[55] Zhang J., He Y., Wang Y., Mao J., Cui X. (2014). Synthesis of a Self-Supporting Faujasite Zeolite Membrane Using Geopolymer Gel for Separation of Alcohol/Water Mixture. Mater. Lett..

[56] Song Y., Li Z., Zhang J., Tang Y., Ge Y., Cui X. (2020). A Low-Cost Biomimetic Heterostructured Multilayer Membrane with Geopolymer Microparticles for Broad-Spectrum Water Purification. ACS Appl. Mater. Interfaces.

